# Effects of Protein Unfolding on Aggregation and Gelation in Lysozyme Solutions

**DOI:** 10.3390/biom10091262

**Published:** 2020-09-02

**Authors:** Shakiba Nikfarjam, Elena V. Jouravleva, Mikhail A. Anisimov, Taylor J. Woehl

**Affiliations:** 1 Department of Chemical and Biomolecular Engineering, University of Maryland, College Park, MD 20742, USA; shnikfar@umd.edu (S.N.); tjwoehl@umd.edu (T.J.W.); 2 Institute for Physical Science and Technology, University of Maryland, College Park, MD 20742, USA; ejouravl@umd.edu

**Keywords:** lysozyme, aggregation, gelation, protein folding/unfolding

## Abstract

In this work, we investigate the role of folding/unfolding equilibrium in protein aggregation and formation of a gel network. Near the neutral pH and at a low buffer ionic strength, the formation of the gel network around unfolding conditions prevents investigations of protein aggregation. In this study, by deploying the fact that in lysozyme solutions the time of folding/unfolding is much shorter than the characteristic time of gelation, we have prevented gelation by rapidly heating the solution up to the unfolding temperature (~80 °C) for a short time (~30 min.) followed by fast cooling to the room temperature. Dynamic light scattering measurements show that if the gelation is prevented, nanosized irreversible aggregates (about 10–15 nm radius) form over a time scale of 10 days. These small aggregates persist and aggregate further into larger aggregates over several weeks. If gelation is not prevented, the nanosized aggregates become the building blocks for the gel network and define its mesh length scale. These results support our previously published conclusion on the nature of mesoscopic aggregates commonly observed in solutions of lysozyme, namely that aggregates do not form from lysozyme monomers in their native folded state. Only with the emergence of a small fraction of unfolded proteins molecules will the aggregates start to appear and grow.

## 1. Introduction

Aggregation of proteins and peptides is a broad and complex phenomenon with several subsets, each progressing via a distinct mechanism [1]. Neurodegenerative diseases such as Alzheimer’s disease and Parkinson’s disease, caused by misfolding and aggregation of proteins followed by precipitation of these aggregates in the central nerves system, are examples of indigenous protein aggregation [2]. Detailed understanding of the aggregation mechanism would help to gain insight into the origin of diseases and their prevention [3]. Because some of the amyloidogenic proteins have a three-dimensional folded structure [4], studying the difference between the driving forces for aggregation of finely folded and partially unfolded proteins would help to elucidate the aggregation mechanisms of pathogenic proteins responsible for amyloid diseases. In biopharmaceuticals protein-based drug formulations may undergo reversible or irreversible aggregation at high concentrations, which negatively affects their therapeutic efficiency and consequently introduces challenges in their production and clinical administration [5]. The increase in the number of protein drugs and the obstacles, caused by aggregation, in production and shelf-life of these drugs is another motivation for studying the aggregation mechanisms [3].

There are several known mechanisms of aggregation in proteins [6]. Depending on the degree of protein folding in the aggregate, the aggregates can be formed either reversible or irreversible [6]. While primary and secondary structures of a protein are internal factors affecting the aggregation propensity of a protein, external factors such as the temperature, pH, ionic strength, shear stress and concentration can also affect the aggregation of proteins in solutions [7,8,9]. The reversible protein oligomers arise from monomers in their native states [10]. As a next step, these oligomers can form irreversible aggregates if the interactions in an oligomeric state favor the conformational change of the monomers towards an aggregate prone state [5,11]. In aggregation through another mechanism, originated from non-native contacts, alteration in conformation of proteins due to heat or physical stress may lead to the formation of irreversible aggregates [6,12,13]. Depending on the pH and ionic strength of solution and at high enough protein concentrations [14,15], by prolong heating, the aggregates associate and eventually can form a gel network [16,17,18].

A clear mechanism of interplay between protein aggregation, phase separation and gelation, is difficult to establish for several reasons—proteins show unique behavior depending on their amino acid content and function. Additionally, aggregation comprises multiple steps depending on the environment. Finally, detection of smaller aggregates with a short lifetime is experimentally nontrivial. Lysozyme is a popular model protein, due to its structural and functional simplicity, extensive documentation and commercial availability. However, there are several unresolved issues regarding lysozyme aggregation. In particular, the nature of its folding/unfolding intermediates, transient oligomerization and the role of gelation.

Several publications of lysozyme solutions mentioned the presence of mesoscopic aggregates or so called “mesoscopic protein-rich clusters” [19,20]. It was found that these aggregates typically had a diameter between 60–200 nm and a fraction of ~10^−4^ of total soluble protein [21]. The aggregates were commonly thought to be reversible [22], undergoing dynamic molecular exchange with protein in solution [23].

In our previous study [24] on the nature of mesoscopic aggregates in solutions of lysozyme, we investigated the effects of concentration, filtration and temperature on the sizes and relative amount of mesoscopic aggregates in solutions of lysozyme. We showed that systematic filtration through 20 nm filters completely removed the aggregates from solution. Moreover, the aggregates did not reemerge. This indicates that the aggregates of lysozyme are unlikely to be a result of reversible self-assembly of unfolded lysozyme molecules. Therefore, the aggregates do not form from lysozyme monomers in their native, biologically active folded state. However, the origin of the main driving forces of aggregation in lysozyme solutions at moderate and low temperatures remain elusive.

In this work we investigate a connection between the formation of lysozyme aggregates and protein unfolding triggered by heating. In particular, we clarify the effect of heating duration on the formation and growth of lysozyme aggregates and a gel network. Formation of the gel network at a nearly neutral pH and low ionic strength occurs in concentrated solutions of lysozyme at about the same temperature as protein denaturation, which prevents direct studies of protein aggregation without being affected by gelation. To block the formation of a gel, we used rapid heating to the protein melting temperature following by quenching to the room temperature. This protocol enabled us to monitor in real time the step-by-step formation of small aggregates, which originate from a certain fraction of unfolded protein molecules, acting as nucleus of amyloid aggregates, by forming amorphous nanoscale aggregates first. If gelation is prevented, nanoscale aggregates (about 10–15 nm radius) are formed. At room temperature, when the samples are monitored for weeks, these clusters persist and can further form larger aggregates. If gelation is not prevented, these clusters become the building blocks of the gel network and define its mesh size.

## 2. Materials and Methods

Lyophilized powder of lysozyme (*M*_w_ = 14 kDa) was obtained from ThermoFisher Scientific (20,000 units/mg solid). HEPES buffer (Sodium salt of 4-(2-hydroxyethyl)-1-piperazineethanesulfonic acid, >99%) was supplied as a solid powder from VWR. All lysozyme samples for dynamic light scattering (DLS) studies were prepared in a 20 mM HEPES buffer at pH 7.8 and filtered through 100 nm and 20 nm pore filters, according to the procedure explained in our previous work [24]. 

By DLS, we studied three concentrations of lysozyme, 18, 30 and 60 mg/mL. The concentration of protein samples were measured by using the absorbance at 280 nm. The details the DLS techniques and measurements are described in our previous work [24]. The lyophilized lysozyme powder was directly dissolved in the buffer for the preparation of the concentrated stock solution. The concentrations were measured after filtration with 200 nm filters. The samples with the desired concentrations were then prepared by dilution. DLS measurements were taken using a multi-angle dynamic light scattering complex from Photocor. Autocorrelation functions were generated for 600 s at each specific angle and temperature. HEPES buffer has a temperature coefficient equal to −0.14, increasing the temperature to 80 °C would decrease the pH less than by 10%, which that should not affect the aggregation. Additionally, a sodium dodecyl sulfate–polyacrylamide gel electrophoresis (SDS-PAGE) gel analysis on both heated and unheated 30 mg/mL samples was performed to investigate the possibility of protein’s degradation. The unheated samples and the sample heated to 80 °C for 30 min were incubated at room temperature for three weeks prior to running the SDS-PAGE gel. Also, the heated and unheated samples were additionally incubated at 95 °C for 5 min right before running the gel.

## 3. Results

### 3.1. Apparent Monomer Size

Primarily, DLS measures the scattering intensity autocorrelation function, exponentially decaying with the diffusive relaxation rate of optical inhomogeneities that exhibit Brownian motion in solution [25],

(1) $$\Gamma =Dq^{2},$$

where *D* is the diffusion coefficient and q=(4π/λ)nsin(θ/2) is the scattering wave number (with *λ* being the wavelength of light, *n* the refractive index, *θ* the scattering angle).

The hydrodynamic radius of inhomogeneities, such as the protein molecules or aggregates, is obtained by assuming the validity of the Stokes-Einstein equation that relates the diffusion coefficient (*D*), viscosity of the solvent (*η*) and hydrodynamic radius *R* [25]:

(2) $$D=\frac{k_{B}T}{6\pi \eta R},$$

where $k_{B}$ is Boltzmann’s constant and *T* is the temperature.

**Figure 1:**
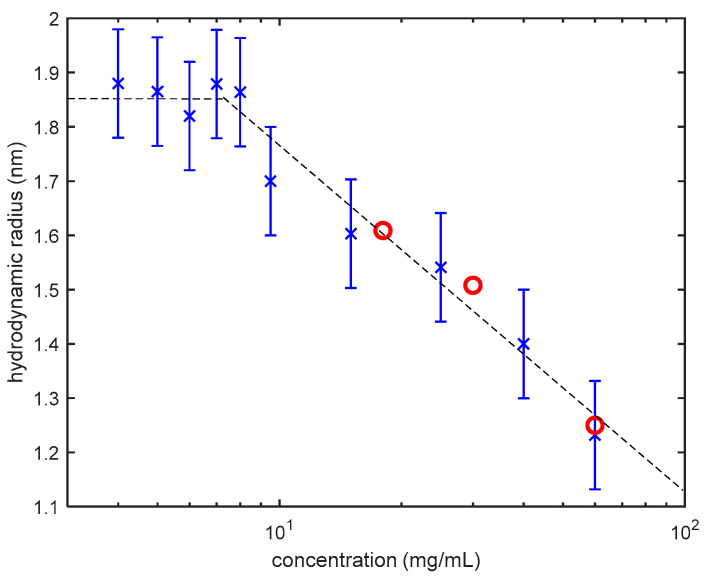
Apparent hydrodynamic radius of lysozyme monomers, obtained by DLS, as a function of concentration at 25 °C [10]. In dilute solutions (<10 mg/mL) it represents the actual size of the lysozyme monomers. Upon the increase of concentration, the apparent radius (blue crosses) sharply decreases because of the interactions between lysozyme molecules. Red circles are the results of our new measurements.

This assumption is fully justified only for dilute solutions of presumably spherical, noninteracting inhomogeneities. Formal application of the Stokes-Einstein equation to semi-dilute and concentrated protein (or, generally, polymer) solutions, results in obtaining a so-called “apparent” hydrodynamic radius that is smaller than the actual hydrodynamic radius of noninteracting individual molecules (moreover, decreasing with the increase of polymer concentration) [26]. The apparent hydrodynamic radius as a function of lysozyme concentration, obtained in our new DLS measurements for three concentrations of lysozyme, is in good agreement with our data reported previously [24]. As demonstrated in Figure 1, the apparent radius corresponds to the actual size of individual molecules (“monomers”) only in dilute solutions of lysozyme (<10 mg/mL). One of the goals in making this chart (Figure 1) was to track changes in sample concentrations using the apparent hydrodynamic radius of lysozyme monomers. During the three weeks of measurements, no change in hydrodynamic radius of lysozyme monomers where observed. This observation confirms that crystal growth and sedimentation did not affect the solution concentrations. This assumption is in agree with the reported phase diagram of lysozyme phase separation and crystallization in 20 mM HEPES and pH of 7.8 reported in Reference [21].

It is important to note that the Stokes-Einstein equation remains valid for mesoscopic lysozyme aggregates even in concentrated solutions. This is because the number of the aggregates are relatively small. Therefore, with respects to the aggregates, the concentrated solution of lysozyme can be regarded as dilute. The hydrodynamic radius of the aggregates thus characterizes their actual size, provided that the actual viscosity of the solution is used in the equation.

### 3.2. Continuous Heating 

**Figure 2:**
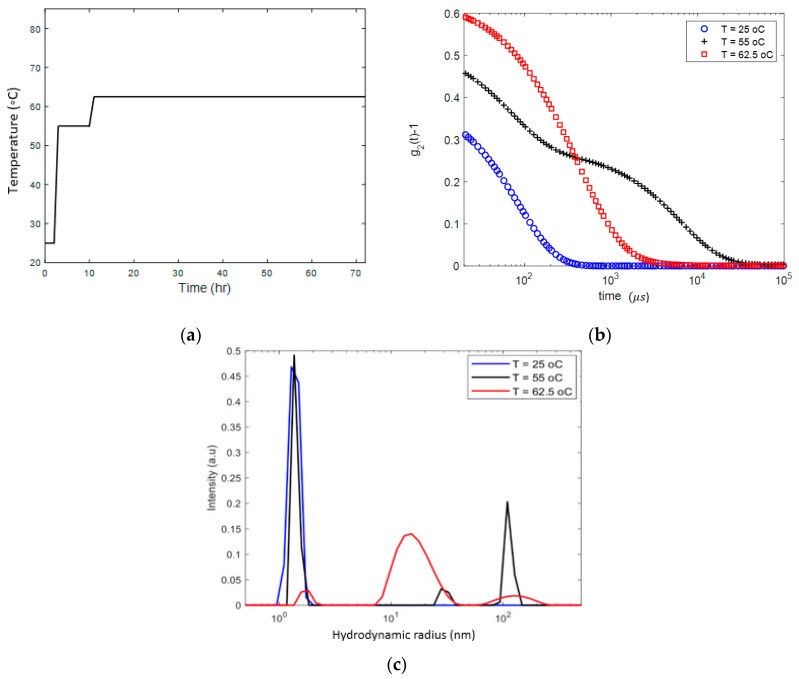
Heating protocol (**a**) and DLS analysis (**b**) and (**c**) demonstrating the process of aggregation and gelation in the 18 mg/mL lysozyme solution, gradually heated to 62.5 °C and kept at this temperature for 60 h. (**b**) DLS autocorrelation function ($\theta =45^{\circ }$) before and after gel formation; (**c**) size distribution of the apparent inhomogeneities before and after gelation.

We first studied an 18 mg/mL lysozyme solution heated from 25 °C to 62.5 °C. Using the protocol outlined in Figure 2a. After holding the solution for 8 h at 55 °C, we observed a change in the DLS autocorrelation function ($g_{2}$), presented in Figure 2b, which marks the onset of aggregation. Two characteristic sizes of the aggregates, of the order of ~30 and ~100 nm, were detected. After further heating and holding the sample for about 60 h at 62.5 °C, as shown in Figure 2b, the autocorrelation function demonstrated a dramatic shift from a double exponential to a broader spectrum of the size distribution (Figure 2c). The distribution reveals three characteristic length scales, one corresponding to apparent size of lysozyme monomers ~1.5 nm, another one to small number of ~100 nm aggregates and a third to inhomogeneities with a length scale of about 15 nm in size, which are dominant. Cooling the sample back to the room temperature did not change the DLS characteristics of the sample. Visual observation showed that the sample was in the gel state. Therefore, we interpret the length scale of about 15 nm as the gelation mesh size.

**Figure 3:**
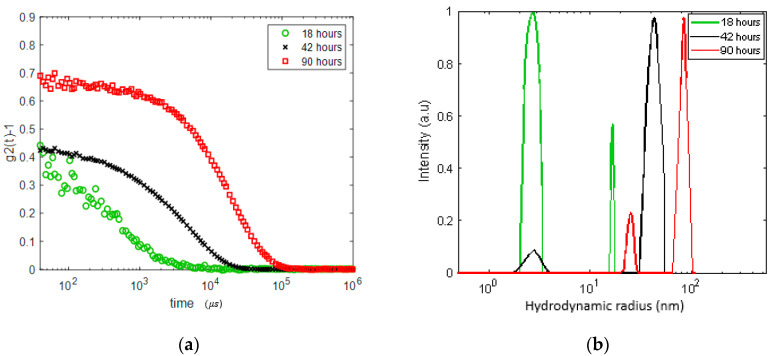
DLS analysis in the 20 mg/mL lysozyme solution gradually heated to 60 °C and kept at this temperature for 18, 42 and 90 h. (**a**) DLS autocorrelation function ($\theta =90^{\circ }$) before and after gel formation; (**b**) size distribution of the apparent inhomogeneities. Note an increase in apparent monomer size due to oligomerization.

We performed another experiment on a lysozyme solution of about the same concentration (20 mg/mL) but with a different protocol. The sample was heated to 60 °C and the DLS correlation function was measured after 18, 42 and 90 h while held at this temperature. The results are presented in Figure 3. We observe the formation of inhomogeneities of a radius growing from ~17 nm after 18 h (with a significant dominance of the nano-sized monomers and oligomers) to ~40 nm after 42 h and to 80–90 nm after 90 h. Remarkably, after 90 h at 60 °C, a meso-size (~20–25 nm) reemerges, while the size distribution of the larger aggregates becomes narrower. Qualitatively, this experiment shares features with the results presented in Figure 2; the growth of aggregates (a faster process) starts before gelation (a slower process of formation of network with a mesh size of about 15–25 nm).

**Figure 4:**
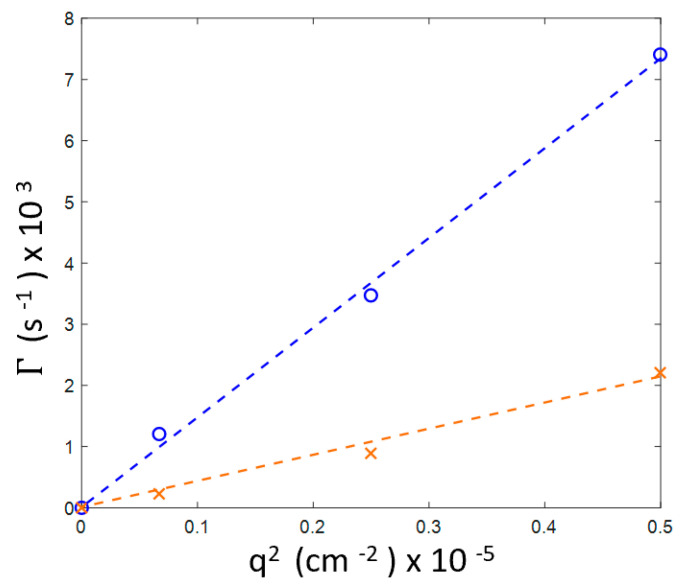
Wave number dependence for three scattering angles (30°, 60° and 90°, q∝sin(θ/2) of the two decay rates in the 20 mg/mL sample after 90 h of holding at 60 °C (see Figure 3b). Blue line: fast mode (smaller aggregates), orange line: slow mode (larger aggregates).

We have also found that, for this sample, despite gelation occurring after 90 h of incubation, the decay rates for the both inhomogeneities, 20–25 and 80–90 nm, exhibit a *q*^2^ linear dependence that is characteristic for diffusive relaxation (Figure 4).

### 3.3. Preventing Gelation and Long-Time Monitoring

**Figure 5:**
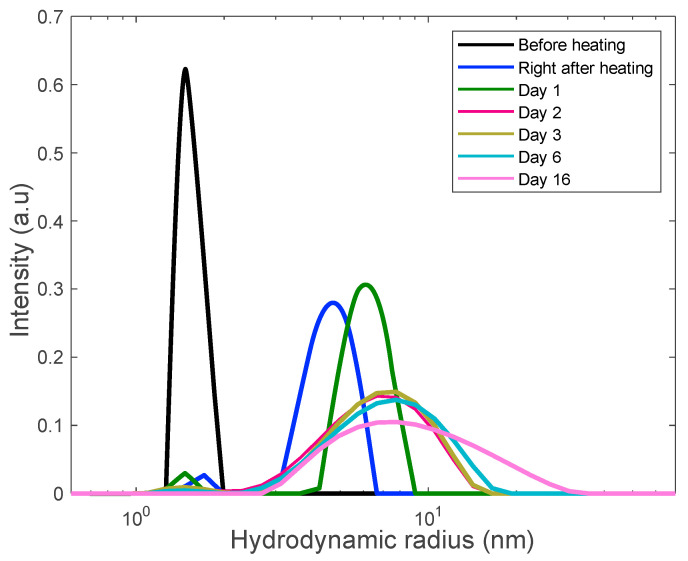
Long-term monitoring of the 18 mg/mL lysozyme solution via DLS. Solution was rapidly heated to the unfolding temperature of 80 °C and held there for 30 min at this temperature to trigger aggregation.

**Figure 6:**
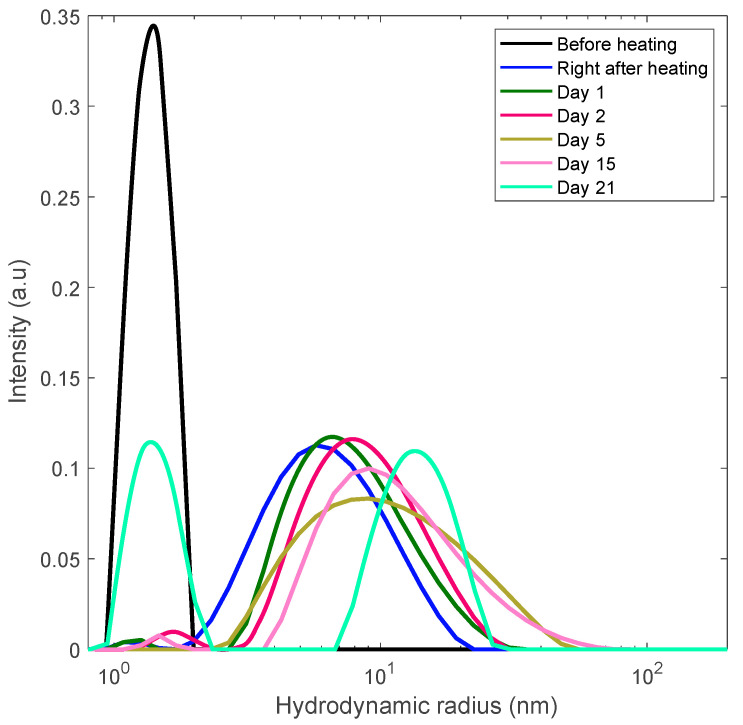
Long-term monitoring of the 30 mg/mL lysozyme solution via DLS. The solution rapidly heated to the unfolding temperature of 80 °C and held there for 30 min at this temperature to trigger aggregation.

Our other heating experiments included the rapid heating of two lysozyme samples (18 mg/mL and 30 mg/mL) to the projected unfolding temperature, 80 °C. We explored the finding that the emergence and growth of aggregates is significantly faster that the time of gelation. To prevent gelation, the sample was kept at this temperature for a short period of time (30 min) following fast cooling to room temperature. The samples were then kept at room temperature and monitored with DLS for over two weeks. The DLS results are shown in Figure 5 and Figure 6.

In these figures the sequence of the line colors corresponds the sequence of monitoring days. One can see the formation of polydisperse nanoscale aggregates with gradual increase of the contribution of aggregates with the size of 10–15 nm. Note that this value is close to the length scale that we interpret as a mesh size of the gel network.

**Figure 7:**
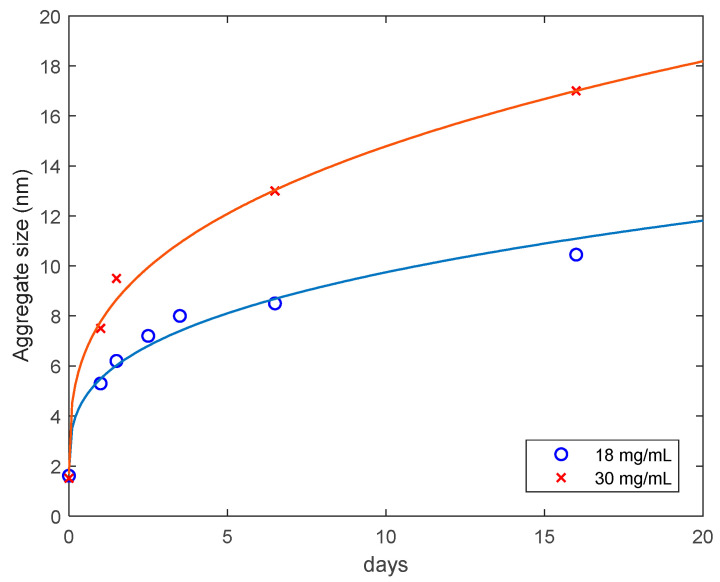
Average hydrodynamic radius of lysozyme aggregates in solutions of 18 mg/mL and 30 mg/mL samples, as a function of time. The solid curves are approximations with Equation (3).

The average size of aggregates for the 18 mg/mL (Sample 1) and 30 mg/mL (Sample 2) lysozyme solutions heated for 30 min in 80 °C, cooled down and monitored at room temperature as a function of the monitoring time is presented in Figure 7. The results are well described by the diffusion-limited aggregation law [27,28]:

(3) $$R=R_{0}+At^{1/d_{f}},$$

where $R_{0}$ is the initial radius of aggregates/monomers (1.6 and 1.5 nm, respectively), *t* is time, A is a constant and $d_{f}$ is the fractal dimension of aggregates (Table 1). For both samples Equation (3) was fitted to the experimental data on the aggregate size as a function of time, with using the MATLAB Levenberg-Marquardt algorithm. The data are consistent with the maximum fractal dimension, $d_{f}=3$, indicating that the lysozyme aggregates can be viewed as relatively compact spherical objects [29]. We find it not surprising that the ratio $A_{2}/A_{1}=1.65$ is almost equal to the sample concentration ratio, namely 1.66.

### 3.4. Formation of Polydisperse Aggregates

**Figure 8:**
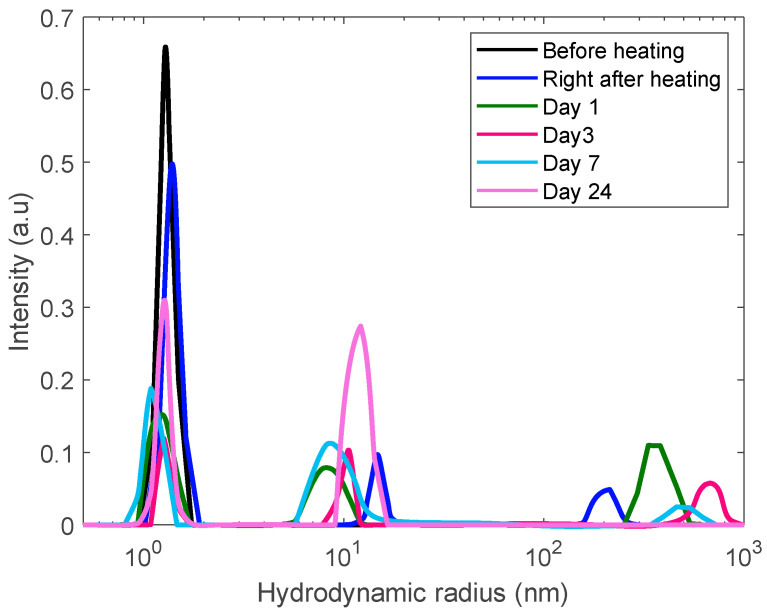
DLS size distribution analysis of a 60 mg/mL lysozyme sample heated for 30 min at 80 °C to trigger aggregation and monitored for 24 days.

We used the same protocol of measurements for a more concentrated solution, 60 mg/mL but observed a different pattern of aggregation phenomena. In addition to monomeric-like and mesoscopic (~10 nm) inhomogeneities, the sample reveals of formation of a broader spectrum of large aggregates growing from 200 nm to almost a micron (Figure 8). However, these aggregates are so large that their growth is accompanied by gradual sedimentation, thus, upon a time, resulting in decreasing their contribution in the DLS correlation function.

**Figure 9:**
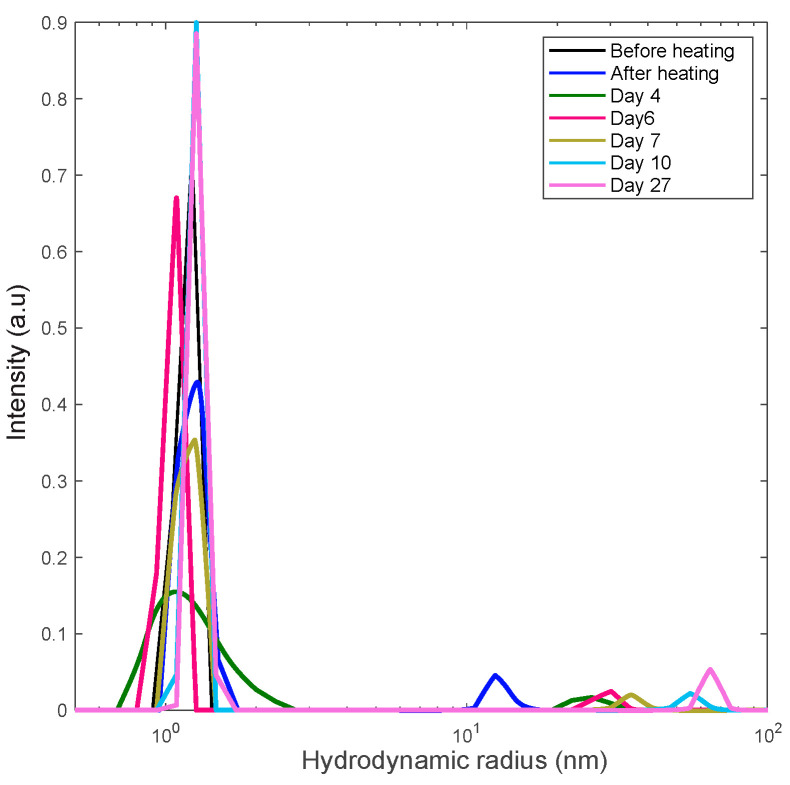
DLS distribution analysis of a 60 mg/mL lysozyme sample heated for 10 min at 80 °C to screen the effect of heating on the aggregation process.

**Figure 10:**
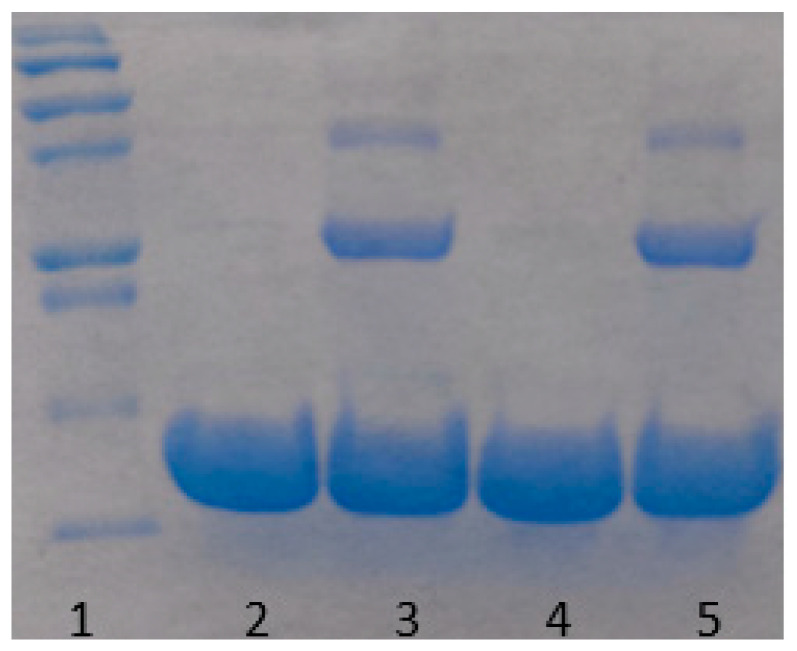
SDS-PAGE gel image for Lysozyme (30 mg/mL) incubated at room temperature for three weeks prior running the gel. Left to right: 1. Ladder (From the bottom 10, 15, 20, 25, 37, 50 kDa). 2. Sample unheated. 3. Sample heated to 80 °C. 4. Sample unheated, then incubated at 95 °C for 5 min prior running the gel. 5. Sample heated to 80 °C, then incubated at 95 °C for 5 min prior running the gel.

Proteins may undergo oxidative or enzymatic degradation over a long time of incubation. This is an important issue that we have considered seriously. Indeed, according to the work of Avanti et al. [30], a heat stressed lysozyme loses about 60% of its activity after 3 weeks of incubation due to degradation. While it is certainly probable that our samples undergo degradation, while being kept at room temperature during a long period of time, we tried to minimize this effect by filtering samples by a 20 nm pore size filter to remove any bacteria from the solution. Buffers were degassed prior to use, so the amount of oxygen in the solution was minimized. However, some degradation was still possible. Therefore, to test the effect of lysozyme’s degradation on the aggregation, a 60 mg/mL sample was kept at 80 °C for 10 min, quickly cooled down to room temperature and was monitored over three weeks. The amount of aggregates formed during that period, was so small that was hardly detectable (Figure 9). Additionally, an SDS-PAGE gel analysis (illustrated in Figure 10) on both heated and unheated 30 mg/mL samples confirmed the absence of protein’s degradation during the experiments, as no lower molecular weight fragments was observed, while some protein dimers and trimers were detected in heated samples. We conclude that the aggregation mechanism in our study is insignificantly affected by lysozyme degradation.

## 4. Discussion

Globular proteins may undergo structural changes due to partial unfolding when being exposed to a higher temperature, even below its true denaturation point. As a result, normally buried hydrophobic residues may act as a crosslinker for intermolecular beta-sheet structures and lead to formation of aggregates of different shapes, globular or amyloid like, depending on the solution conditions [16,31,32]. Continuous heating leads to association of aggregates and subsequent formation of a gel network [16,17,18,32,33].

Based on our findings, we can qualitatively suggest a mechanism of aggregation that we observed in lysozyme samples treated under different heating protocols. Lysozyme at low ionic strength (20 mM) and nearly neutral pH unfolds at a temperature around 80 °C [22]. 

We estimate the fraction of unfolded lysozyme molecules, ϕ, as a function of temperature through the enthalpy of folding/unfolding, $\Delta H_{0}$ (kJ/mol), at the “melting” temperature $T_{0}$, defined to be the temperature at which the fraction of folded and unfolded lysozyme is equal. In the first approximation, we obtain:

(4) $$\frac{\Delta H_{0}}{k_{B}}\left(\frac{1}{T}-\frac{1}{T_{0}}\right)=\ln\frac{\phi }{1-\phi }.$$

**Figure 11:**
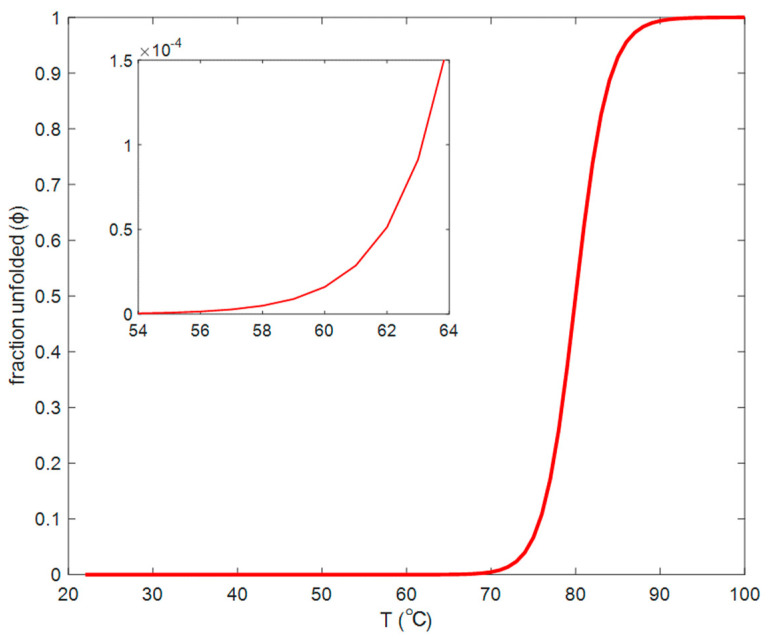
Fraction of unfolded lysozyme as a function of temperature.

According to References [22,34,35], the enthalpy of lysozyme unfolding varies between 200 and 600 kJ/mol, depending on experimental techniques and buffer conditions. In Figure 11 we present the fraction of unfolded lysozyme, calculated from Equation (4), with adopted values of $\Delta H_{0}=540\;\mathrm{kJ}/\mathrm{mol}\;\mathrm{and}\;T_{0}=80{\;}^{\circ }C$ [34]. In this adoption we neglect a dependence of $\Delta H_{0}\;\mathrm{and}\;T_{0}$ on lysozyme concentration.

In the hierarchy of times scales, the lysozyme interconversion rate is fastest (overall folding time is of the order of 1 s) [36,37], while characteristic times of the motion of molecular segments could be of the order of 1 μs [38]. Then a relatively lower rate of aggregation follows. The rate of gelation is the slowest and strongly depends on concentration and temperature. Our experiments on a 20 mg/mL sample showed that at 60 °C it took up to 90 h to observe a distinct gel state. The lower the temperature, the smaller the fraction of unfolded lysozyme molecules, the smaller the probability of aggregation and larger the time of gelation. As estimated with Equation (4), the fraction of the unfolded molecules at 60 °C could be as small as $2\times 10^{-3}$%. Nevertheless, these molecules serve as seeds of nucleation to form growing aggregates and eventually a gel network. We must note that even an extremely small number of mesoscopic aggregates could make a significant contribution to the DLS correlation function because light scattering intensity strongly depends on the size, especially at small scattering angles. The reaction coordinate, the fraction of unfolded protein molecules, exhibit thermal fluctuations around its “chemical reaction” equilibrium value. The average amplitude and lifetime of these fluctuations depend on the thermodynamic conditions and can be found by statistical thermodynamics. However, because the distribution of the fluctuations in space and time is Gaussian, occasionally, during a long observation time, the deviations in concentration of the unfolded protein from the equilibrium value, could become significant and hence initiate the gelation process.

The analysis of the correlation function of gel formed by heating lysozyme solutions ~20 °C below the melting temperature over a long period of time indicates the presence of diffusion relaxation in the gel. In this work we did not address the interesting dynamic properties of gels, which would require a more detailed DLS experiments and analysis. We just note that the correlation function of density/concentration fluctuations in gel can exhibit a compressed exponential decay [16,33]. The observed relaxation modes can be attributed to concentration fluctuations of the aggregates which are not a part of gel network, branching points, segments and restructuring of the gel [31,32,39]. In case of the extremely fragile lysozyme gel, the diffusive inhomogeneities with the sizes ~15–20 and ~100 nm (Figure 2c, red curves), could be interpreted as a branch/mesh pattern forming the gel network (smaller size) and separate aggregates (larger size) diffusing across the network. 

## 5. Conclusions

In this work, the role of equilibrium unfolding as the main driving force for protein aggregation at elevated temperatures is clarified. While monomeric lysozyme molecules do not have a propensity to form detectable aggregates for a long period of time [24], increasing the temperature and shifting the equilibrium to marginally unfolded protein leads to the emergence of aggregate seeds. These seeds can either rapidly grow if heated continuously or undergo a slow diffusion limited aggregation over a long time of monitoring.

Our results resonate with the observations of previous investigators on aggregation phenomena in lysozyme solutions. The importance of driving forces for aggregation at physiological temperatures was emphasized in the work of Vekilov et al. [23], where the presence of transient dimers of lysozyme was found. Also, according to Strander et al. [10], at the same ionic strength and pH, the presence of short-range attractions and long-range repulsions leads to the formation of dense liquid phases in lysozyme solutions. The reported clusters have an aggregation number between 2–7 monomers per cluster at the range of concentrations of 50–100 mg/mL. DLS was not able to detect the presence of such clusters. The lack of DLS ability for the detection of these clusters can be because of the short lifetime of the clusters and/or their small population. However, although these clusters have a very small population, they may be involved in the formation of larger observed aggregates.

According to References [40,41,42], amyloid aggregates can eventually form a viscoelastic gel solution. Since the time scale of gel formation is much longer than the folding/unfolding interconversion and aggregation, the aggregation is a required step for the gel formation. A fundamental, yet unexplored problem that could be addressed in further experimental and theoretical studies, would be a possible role of fluctuations of unfolded molecular states near the interconversion equilibrium. Protein molecules can fluctuate between their alternative states and eventually bind to each other and form aggregates. Fluctuation-induced aggregation [43] and, more recently, fluctuation-induced phase transitions [44] have been discussed with respect to the phase behavior of classical colloids. It would be interesting to investigate this effect with respect to aggregation of proteins. 

## Figures and Tables

**Table 1:** Fitting parameters in the diffusion-limited aggregation law, with $d_{f}=3$.

Concentration (mg/mL)	Amplitude (A)	Standard Deviation
18	3.68	±0.37
30	6.07	±0.36
